# Aldosterone from endometrial glands is benefit for human decidualization

**DOI:** 10.1038/s41419-020-02844-9

**Published:** 2020-08-13

**Authors:** Shu-Yun Li, Zhuo Song, Ya-Ping Yan, Bo Li, Min-Jie Song, Yue-Fang Liu, Zhen-Shan Yang, Meng-Yuan Li, Ai-Xia Liu, Song Quan, Zeng-Ming Yang

**Affiliations:** 1 College of Veterinary Medicine, South China Agricultural University, Guangzhou, 510642 China; 2 Department of Reproductive Endocrinology, Women’s Hospital, School of Medicine, Zhejiang University, Xueshi Road, Hangzhou, 310006 China; 3 Center for Reproductive Medicine, Nanfang Hospital, Southern Medical University, Guangzhou, 510515 China

**Keywords:** Reproductive biology, Obesity

## Abstract

Local renin-angiotensin system (RAS) in female reproductive system is involved in many physiological and pathological processes, such as follicular development, ovarian angiogenesis, ovarian, and endometrial cancer progress. However, studies on the functional relevance of RAS in human endometrium are limited, especially for renin-angiotensin-aldosterone system (RAAS). In this study, we defined the location of RAS components in human endometrium. We found that angiotensin II type-1 receptor (AT_1_R) and aldosterone synthase (CYP11B2), major components of RAAS, are specifically expressed in endometrial gland during mid-secretory phase. Aldosterone receptor, mineralocorticoid receptor (MR), is elevated in stroma in mid-secretory endometrium. In vitro, MR is also activated by aldosterone during decidualization. Activated MR initiates LKB1 expression, followed by phosphorylating of AMPK that stimulates PDK4 expression. The impact of PDK4 on decidualization is independent on PDHE1α inactivation. Based on co-immunoprecipitation, PDK4 interacts with p-CREB to prevent its ubiquitination for facilitating decidualization via FOXO1. Restrain of MR activation interrupts LKB1/p-AMPK/PDK4/p-CREB/FOXO1 pathway induced by aldosterone, indicating that aldosterone action on decidualization is mainly dependent on MR stimulation. Aldosterone biosynthesized in endometrial gland during mid-secretory phase promotes decidualization via activating MR/LKB1/p-AMPK/PDK4/p-CREB/FOXO1 signaling pathway. This study provides the valuable information for understanding the underlying mechanism during decidualization.

## Introduction

Successful pregnancy requires embryo implantation into a receptive endometrium and subsequent decidualization in rodents and primates^1,2^. Human reproduction is remarkably inefficient, with only 13–14% chance for natural conception per menstrual cycle^2^. Moreover, nearly 30% of pregnancy ends in pregnancy loss^3,4^. Epithelial glands are essential for establishment of uterine receptivity and stromal decidualization^2,5,6^. Decidualization is one indispensable process during successful pregnancy^1,2^. Defective decidualization can result in preeclampsia or miscarriage^2,7^. Unlike rodents, human decidualization spontaneously initiates during the mid-secretory phase^8^.

The renin-angiotensin-aldosterone system (RAAS) is a peptidergic system with endocrine characteristics^9^. Angiotensinogen (AGT) is cleaved by renin to form a decapeptide angiotensin 1 (ANG1), which is then activated into ANG2 by angiotensin converting enzyme (ACE)^9,10^. ANG2 interacts with its specific and high-affinity receptors (AT1R and AT2R) on the surface of target cells to exert its biological effects^11^. AT1R is involved in aldosterone production by up-regulating cytochrome P450 family 11 subfamily B member 2 (CYP11B2), an aldosterone synthase^12^. Aldosterone, a steroid hormone secreted by adrenal cortex, is the principle mineralocorticoid controlling sodium and potassium balance^12^. Aldosterone can bind to the mineralocorticoid receptor (MR) and induce several target genes^13,14^. MR has an equal affinity for aldosterone and cortisol^15^. 11β-hydroxysteroid dehydrogenase type 2 (HSD11β2) plays a key role in MR specificity for aldosterone through converting cortisol into the inactive metabolite cortisone^15,16^.

Locally active renin-angiotensin system (RAS) has been reported in human decidua^17^. Renin and ACE are localized in human endometrial epithelium throughout the menstrual cycle^18^. Both ANG2 and AT1R are expressed in human endometrium through the menstrual cycle^19^. It seems that local RAAS is active in human endometrium. However, its specific effects remain unclear.

Pyruvate dehydrogenase kinases (PDKs) inactivate pyruvate dehydrogenase complex (PDC) that catalyzes oxidative decarboxylation of pyruvate by phosphorylation of pyruvate dehydrogenase E1 alpha 1 subunit (PDHE1α)^20^. PDK4 is regulated in a PGC-1α-independent manner^21–23^. Phosphorylation of PDHE1α compromise PDC activity, leading to restriction of pyruvate oxidation and accelerating glycolysis^24^. In mice, Warburg-like glycolysis is beneficial to decidualization^25^. The flux of glucose is increased during human decidualization^26^.

During the secretory phase of menstrual cycle, morphological differentiation of glandular and luminal epithelia accompanies the induction and secretion of proteins into uterine lumen for the preparation of embryo implantation^27^. Uterine glands contribute not only for implantation but also for decidualization^5^. Leukemia inhibitory factor (LIF) secreted from uterine glands is important for decidualization in mice^5^. However, how uterine glands regulate decidualization are still poorly defined. In this study, we discovered the presence of local RAAS in glandular epithelium during the mid-secretory phase of menstrual cycle and found that aldosterone activates LKB1-p-AMPK-PDK4-p-CREB-FOXO1 signaling during decidualization.

## Materials and methods

### Human Ishikawa endometrial carcinoma cells

Ishikawa endometrial adenocarcinoma cells line was purchased from cell bank of Chinese Academy of Science (Shanghai, China). ECC1 cell line was purchased from ATCC (CRL-2923). Ishikawa and ECC1 cells were routinely cultured in Dulbecco’s Modified Eagle’s Medium/Nutrient Mixture F-12 (DMEM/F-12 media, D2906, Sigma-Aldrich, St. Louis, MO) containing 10% fetal bovine serum (FBS, 04–001–1A, Biological Industries, Cromwell, CT), 100 units/ml penicillin and 0.1 mg/ml streptomycin (Penicillin-Streptomycin, 15140-122, Gibco, Grand Island, NY). Cells were cultured at 37 °C in a humidified atmosphere of 5% CO_2_.

### Collection of human endometrial samples

Human endometrial samples were collected by biopsy from normally cycling 25–40 years old women with informed consent in Nanfang Hospital (Guangzhou, China). Endometrial samples were dated for menstrual cycle phase according to endometrial morphology and menstrual history^28^. All human procedures regarding endometrial biopsies were approved by the Institutional Committee on the Use of Human Subjects of Nanfang Hospital. Decidual tissues were obtained from 31–38 years old women undergoing elective terminations of first-trimester pregnancy in Women’s Hospital (Hangzhou, China). All human procedures regarding for decidual tissues were approved by Ethical Committee of Women’s Hospital, School of Medicine, Zhejiang University. In total, six endometrial biopsies for each menstrual cycle phase and five decidua tissues were used in this study.

### Isolation of human endometrial stromal cells and decidualization in vitro

Human endometrial stromal cells were isolated as previously described^29^. Briefly, endometrial tissues were rinsed in DMEM/F12 medium and minced. Minced tissues were incubated with collagenase type I (134 U/ml) (Sigma-Aldrich, St. Louis, MO) and deoxyribonuclease type 1 (156 U/ml) (Roche, Basel, Switzerland) in 10 ml DMEM/F12 for 1 h at 37 °C with manual agitation at a 20 min interval. After digestion, tissues were filtered through 40 μm cell strainers to eliminate glandular clumps and flushed with DMEM/F12 containing 10% certified FBS (cFBS, 04-0201-1A, Biological Industries, Cromwell, CT). Cells were collected by centrifugation (1000 × *g*, 5 min) and re-suspended in DMEM/F12 medium containing 10% cFBS for culture. The medium was changed 6–18 h post-seeding to remove unattached epithelial cells, red blood cells and immune cells.

Cells were cultured with 250 μM dibutyryl cyclic adenosine monophosphate (db-cAMP; Sigma-Aldrich) and 1 μM medroxyprogesterone acetate (MPA) to induce decidualization in vitro as previously described^30^. Cells were harvested for further analysis after 6 days of treatment.

### siRNA transfection

Human stromal cells were transfected with each siRNA (100 pM) by Lipofectamine 2000 (Invitrogen, Carlsbad, CA) as to the manufacturer’s instruction. The siRNA oligonucleotides targeting for PDK4 and PDHE1α were designed and synthesized by Ribobio Co, Ltd (Guangzhou, China). Cells were collected 4 days after treatment for real-time PCR or for Western blot assay.

### RNA extraction and real-time PCR

Total RNAs were extracted using RNAiso Plus (9109, Takara, Tokyo, Japan), followed by quantification and quality assessment as previously described^31^. Total RNAs were reverse transcribed using the PrimeScript reverse transcriptase reagent kit (Perfect Real Time, RR037A, TaKaRa, Tokyo, Japan). The conditions used for real-time PCR were as follows: 95 °C for 10 s followed by 39 cycles of 95 °C for 5 s and 60 °C for 34 s. All reactions were run in triplicate. RPL7 was used for normalization. Data from real-time PCR were analyzed using the ΔΔCt method^32^. Primers used for real-time PCR and sequences used for siRNA were listed in Table S1.

### Western blot

Total cellular proteins were extracted using lysis buffer (50 mM Tris-HCl, pH 7.4), 150 mM NaCl, 5 mM EDTA, 10 mM NaF, 1 mM Na3VO3, 1% Sodium deoxycholate, 1% Triton X-100, and 0.1% SDS) with complete protease inhibitor cocktail (4693116001, Roche, Basel, Switzerland). Subcellular nuclear fractionation was isolated as previously described^31^. Protein concentration was measured by BCA Reagent kit (23225, Thermo Fisher Scientific, Waltham, MA).

Tetrameric Pkm2 was analyzed as previously described^25^. Briefly, the protein lysate was extracted by TG buffer (20 mM HEPES, pH 7.5, 1% Triton X-100 and 10% glycerol) and incubated with 0.01% glutaraldehyde for 5 min. The reaction was terminated by adding 1 M Tris buffer (pH 8.0). Protein lysates (10 μg) were electrophoresed using 10% SDS-PAGE gel and transferred onto polyvinylidene fluoride (PVDF) membranes (IPVH00010, Millipore, Billerica, MA). After blocking in 5% nonfat dry milk (A600669, Sangon, Shanghai, China) for 1 h, membranes were probed with the corresponding primary antibodies overnight at 4 °C. After washing, membranes were incubated with matched secondary antibodies conjugated with horseradish peroxidase for 1 h and visualized using ECL chemiluminescent kit (32106, Thermo Fisher Scientific). The primary antibodies used were as follows: mouse anti-RENIN (1:500, SC-137252, Santa Cruz, Dallas, TX), rabbit anti-ACE (1:1000, ab75762, Abcam, Cambridge, UK), rabbit anti-AT1R (1:1000, 25343-1-AP, Proteintech, Rosemont, IL), rabbit anti-CYP11B2 (1:1000, 20968-1-AP, Proteintech), rabbit anti-MR (1:1000, 21854-1-AP, Proteintech) and PDK4 (1:1000, AP7041B, Abgent), rabbit anti-LKB1 (1:1000, 3050s, Cell Signaling Technology, Danvers, MA), rabbit anti-p-AMPK (1:1000, 2535 s, Cell Signaling Technology), rabbit anti-p-CREB (1:1000, 9198s, Cell Signaling Technology), rabbit anti-CREB (1:1000, 9197s, Cell Signaling Technology), rabbit anti-LAMIN A/C (1:1000, 2032s, Cell Signaling Technology), rabbit anti-PKM2 (1:1000, 4053s, Cell Signaling Technology), rabbit anti-LDHA (1:1000, 3582s, Cell Signaling Technology), mouse anti-PDH (1:1000, SC-377092, Santa Cruz), and rabbit anti-FOXO1 (1:1000, 2880s, Cell Signaling Technology). β-actin is used for an internal control.

### Co-immunoprecipitation

The human endometrial stromal cells under in vitro decidualization for 6 days were used for co-immunoprecipitation. Co-immunoprecipitation was performed with rabbit anti-p-CREB antibody and rabbit IgG as a control as previously described^33^. Western blot was performed using anti-PDK4 and anti-p-CREB antibodies, respectively.

### Immunohistochemistry

Human paraffin-embedded uterine sections (5 μm) were deparaffinized and rehydrated. Antigen retrieval was performed in 10 mM sodium citrate buffer (pH 6.0) by microwaving for 10 min and then cooling to room temperature. The antigen retrieval for AT1R antibody was performed in 1 mM EDTA (pH 9.0). Endogenous horseradish peroxidase (HRP) activity was inhibited with 3% H_2_O_2_ for 15 min. Nonspecific binding was blocked with 10% horse serum at 37 °C for 60 min. Sections were incubated with each primary antibody diluted with 10% horse serum in PBS at 4 °C overnight. The primary antibodies used in this study included mouse anti-RENIN (1:100, SC-137252, Santa Cruz), rabbit anti-ACE (1:100, ab75762, Abcam), rabbit anti-AT1R (1:75, 25343-1-AP, Proteintech), rabbit anti-CYP11B2 (1:50, 20968-1-AP, Proteintech), anti-IGFBP1 (1:200, SC-55474, Santa Cruz), anti-CK18 (1:200, SC-6259, Santa Cruz), rabbit anti-MR (1:400, 21854-1-AP, Proteintech) and PDK4 (1:200, ap7041b, Abgent). Normal rabbit IgG (1:200, 2729s, Cell Signaling Technology) or normal mouse IgG (1:200, SC-2025, Santa Cruz) was used as a negative control. After washing and incubating with biotinylated rabbit anti-mouse IgG or goat anti-rabbit IgG (1:200, Zhongshan Golden Bridge, Beijing, China) for 30 min, sections were incubated with streptavidin-HRP complex (1:200, Zhongshan Golden Bridge) for 30 min. The signals were visualized using DAB Horseradish Peroxidase Color Development Kit according to the manufacturer’s protocol (Zhongshan Golden Bridge).

### Immunofluorescence

Human endometrial stromal cells cultured on cover glass were fixed in 4% paraformaldehyde (158127, Sigma-Aldrich) diluted in PBS for 10 min at 4 °C and washed three times in PBS. After treatment with 0.1% Triton X-100 in PBS for 20 min, sections were blocked with 5% donkey serum for 1 h at 37 °C, and incubated with anti-MR antibody (1:200, 21854-1-AP, Proteintech) overnight at 4 °C. After washing in PBS, sections were incubated with secondary antibody (711-225-152, Jackson ImmunoResearch, West Grove, PA) for 30 min at 37 °C and counterstained with 4, 6-diamidino-2-phenylindole dihydrochloride (DAPI, Zhongshan Golden Bridge). The fluorescent signals were examined under a fluorescence microscopy. Negative control slides were incubated with rabbit IgG (Santa Cruz).

### Assay of aldosterone

Aldosterone was assayed in cultured cells and cultured medium samples by aldosterone ELISA kit as to manufacturer’s instructions (Cayman, 501090). The volume of cultured medium was 100 μl in each sample and the cell number is 1 × 10^5^ cells in each sample.

### Statistical analysis

Data were presented as the mean ± standard deviation (SD) unless stated otherwise. The significance of difference between two groups was assessed by Student’s *t*-test. One way ANOVA test was used for the comparisons of multiple groups. *P* value < 0.05 was considered statistically significant. *P* values < 0.05, *P* values < 0.01, and *P* values < 0.001 were shown with */#, **/##, and ***/###, respectively.

## Results

### Expression of RAAS family members in human endometrium during the menstrual cycle

**Fig. 1:**
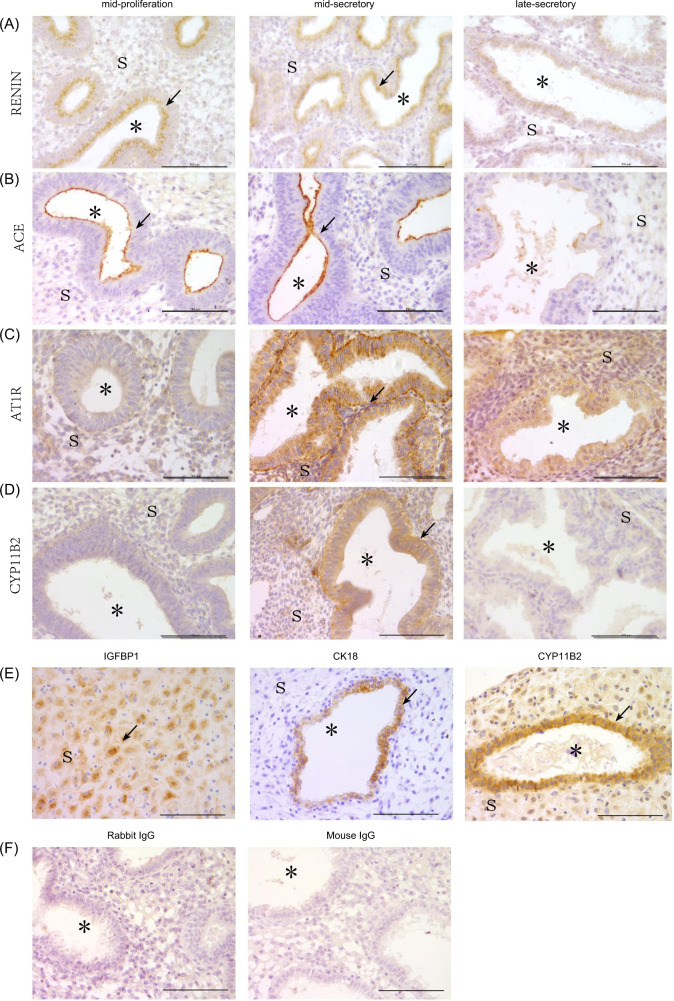
Immunostaining analysis of renin-angiotensin-aldosterone system in human endometrium during menstrual cycle and pregnancy. **a** RENIN immunostaining during menstrual cycle. **b** ACE immunostaining during menstrual cycle. **c** AT1R immunostaining during menstrual cycle. **d** CYP11B2 immunostaining during menstrual cycle. **e** IGFBP1, CK18, and CYP11B2 immunostaining in pregnant decidua. **f** Negative control with rabbit or mouse IgG as a replacement for each primary antibody. Arrows indicate glandular epithelium; Scale bar = 100 μm; * glandular lumen; S stromal cells.

Immunohistochemistry was used to analyze the expressions of RENIN, ACE, AT1R and CYP11B2 in human endometrium. RENIN and ACE were specifically expressed in glandular epithelium in mid-proliferative and mid-secretory phases, but weakly in late-secretory phase (Fig. 1a, b). AT1R was strongly expression in glandular epithelium in mid-secretory phase, while weakly in late-secretory phase and undetectable in mid-proliferative phase (Fig. 1c). CYP11B2, an aldosterone synthase, was weakly located in glandular epithelium in mid-secretory phase (Fig. 1d). In the pregnant decidua, CYP11B2 was strongly expressed in glands, and is also weakly expressed in stromal cells, in which decidualization was verified by IGFBP1 expression, and uterine glands were confirmed by cytokeratin 18 (CK18) expression (Fig. 1e). There were no detectable signals in negative controls incubated with rabbit IgG or mouse IgG (Fig. 1f). These results suggested that a local RAAS should be present in human endometrium during mid-secretory phase, and aldosterone can be synthesized in glandular epithelium in pre-decidua and decidua.

### Progesterone induces aldosterone release in cultured epithelial cells

**Fig. 2:**
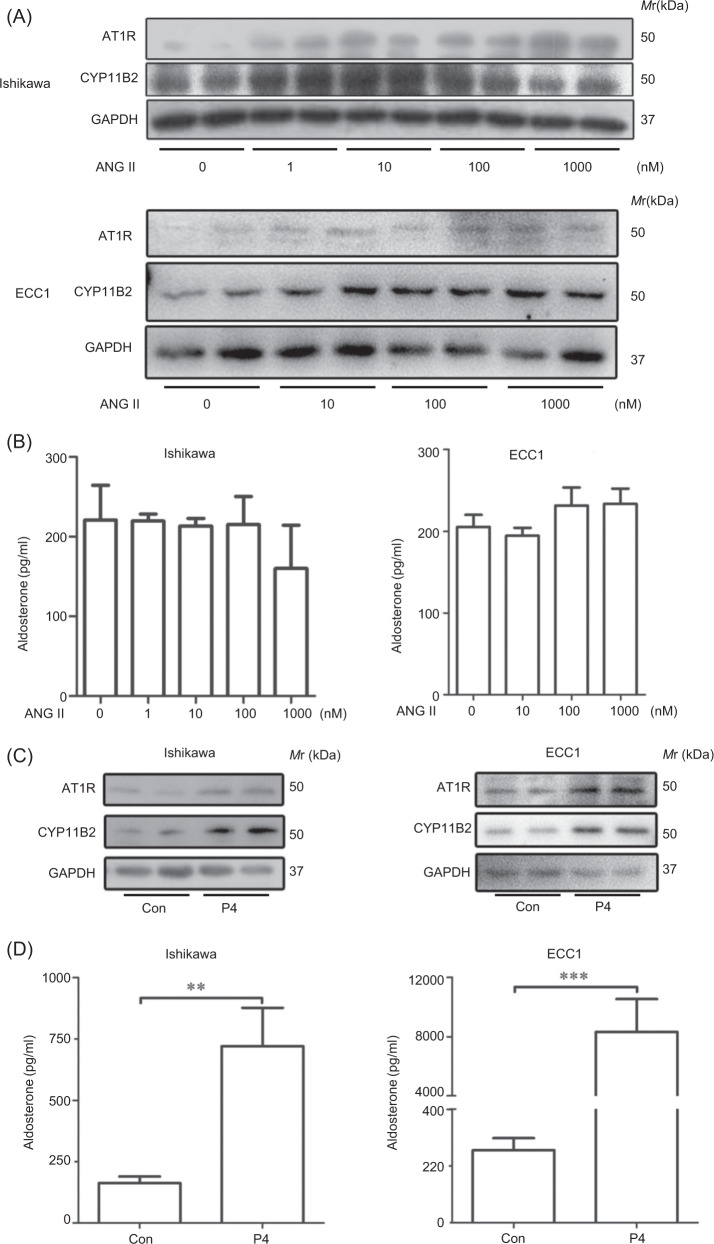
Progesterone stimulates aldosterone secretion in vitro. **a** Western blot analysis of AT1R and CYP11B2 protein levels after Ishikawa cells and ECC1 cells were treated with ANG II. **b** Aldosterone amount in cultured medium. **c** Western blot analysis of RENIN, ACE, AT1R, and CYP11B2 proteins after Ishikawa cells and ECC1 cells were treated with P4. **d** Aldosterone levels in cultured medium. GAPDH was used as internal control. ANG II angiotensin 2; P4 progesterone.

Ishikawa cell and ECC1 cell lines were used to further examine the effect of ANG 2 on AT1R and CYP11B2 protein expression in glandular epithelium. Both AT1R and CYP11B2 protein levels were increased after Ishikawa cells were treated with 1, 10, 100, and 1000 nM ANG2 for 48 h, respectively (Fig. 2a). These results indicated ANG2 stimulates AT1R and CYP11B2 expressions in glandular epithelium.

However, aldosterone concentration in cultured medium did not increase after Ishikawa or ECC1 cells were treated with ANG2 for 48 h (Fig. 2b). These data indicated that even if protein levels of aldosterone synthase were up-regulated following ANG2 treatment in vitro, aldosterone synthesize was not detected without its precursor. Progesterone is the precursor of aldosterone^34^. Ishikawa and ECC1 cells were treated with 1 μM progesterone to explore whether progesterone could induce aldosterone release in vitro. Western blot analysis showed that progesterone could increase AT1R and CYP11B2 protein levels in both Ishikawa and ECC1 cells (Fig. 2c). Aldosterone levels were significant elevated after Ishikawa and ECC1 cells were treated with progesterone for 48 h (Fig. 2d). Our data suggested progesterone, as the precursor of aldosterone, can promote aldosterone synthesis via activating RAAS in vitro.

### Aldosterone promotes stromal decidualization via p-CREB/FOXO1

**Fig. 3:**
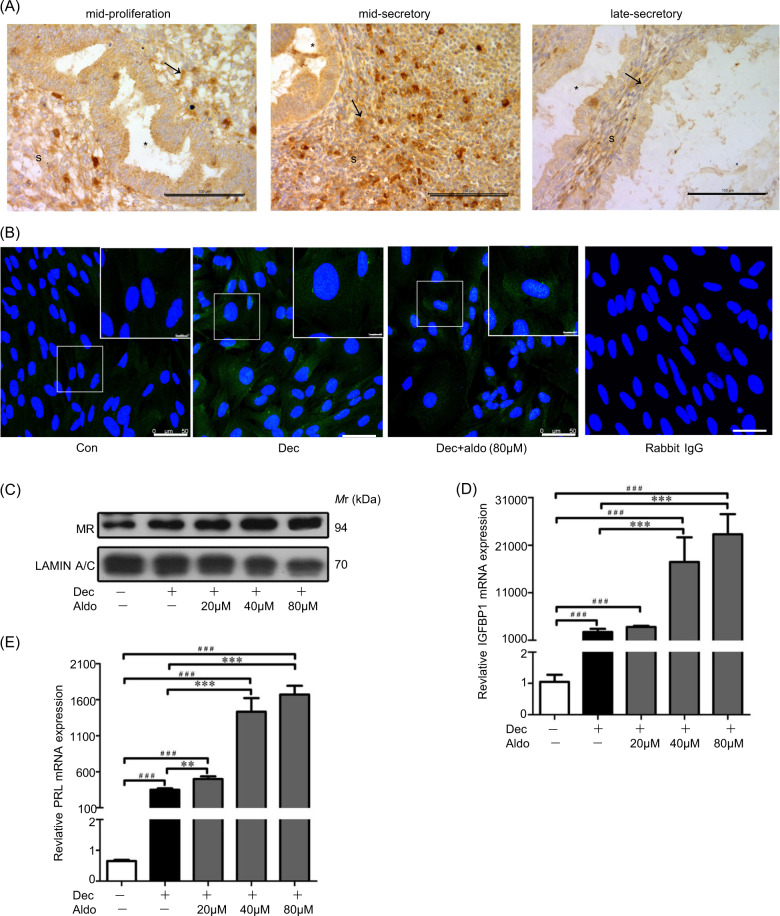
Aldosterone stimulates in vitro decidualization through MR. **a** MR immunostaining in human endometrium during the menstrual cycle. Scale bar = 100 μm; * glandular lumen; S stromal cells. **b** MR immunofluorescence under in vitro decidualization after treatment with aldosterone. Bar = 50 μm. **c** Western blot analysis on effects of aldosterone on nuclear MR. **d** Effects of aldosterone on IGFBP1 mRNA levels in decidual cells. **e** Effects of aldosterone on PRL mRNA levels in decidual cells. LAMIN A/C as nuclear control.

MR protein was mainly located in stromal cells and highly expressed during mid-secretory phase (Fig. 3a). Under in vitro decidualization, MR protein level was up-regulated (Fig. 3b). To verify whether aldosterone can activate MR, decidual cells were treated with different concentrations (20, 40, and 80 μM) of aldosterone. MR protein was localized in the nuclei of some decidual cells treated with 20 μM aldosterone (data not shown). The MR amount in nuclei was dependent on aldosterone dose. When decidual cells were treated with 80 μM aldosterone, MR protein enriched in the nuclei of most decidual cells (Fig. 3b). No detectable signals were detected in negative controls incubated with rabbit IgG or mouse IgG (Fig. 3b). Western blot also showed that MR protein level in nuclei was increased by aldosterone treatment (Fig. 3c). The induction of IGFBP1 and PRL mRNA levels in aldosterone-treated group revealed that aldosterone promoted decidualization (Fig. 3d, e).

**Fig. 4:**
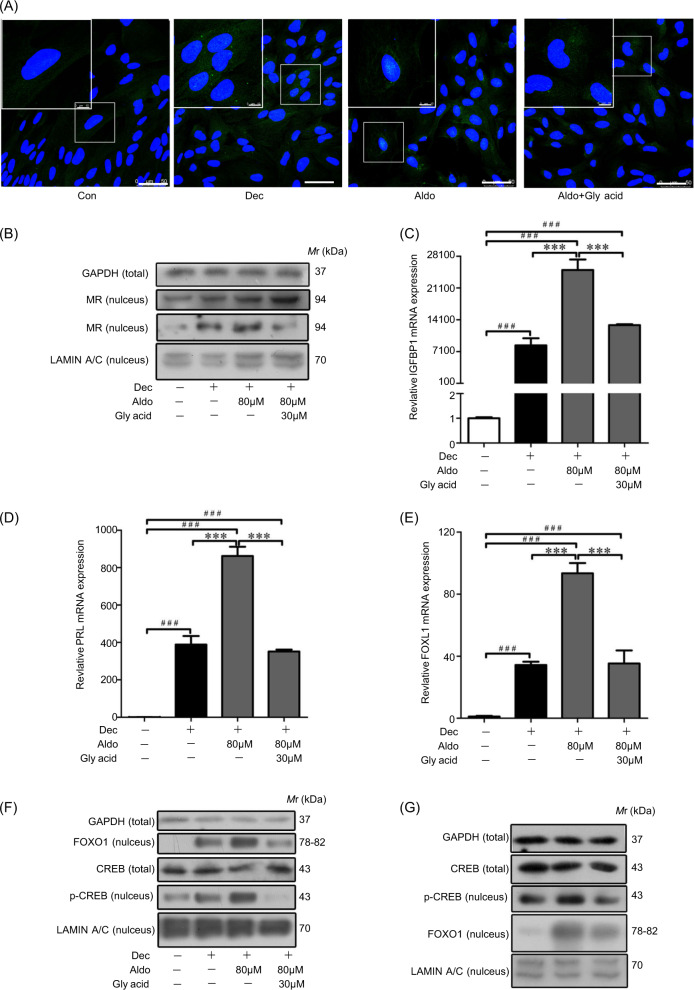
Aldosterone regulates decidualization through FOXO1. **a** Immunofluorescent analysis of MR protein after decidual cells were treated with aldosterone and glycyrrhetic acid (Gly acid), respectively. **b** Western blot analysis of cytoplasmic and nuclear MR protein levels after decidual cells were treated with aldosterone and glycyrrhetic acid. **c** Effects of aldosterone and glycyrrhetic acid on IGFBP1 expression. **d** Effects of aldosterone and glycyrrhetic acid on PRL expression. **e** Effects of aldosterone and glycyrrhetic acid on FOXO1 expression. **f** Western blot analysis of CREB, FOXO1, and p-CREB protein levels in nuclear fraction of decidual cells. **g** Western blot analysis on effects of CREB inhibitor (KG-501) on CREB, nuclear FOXO1, and p-CREB. RPL7 was used as internal control for real time PCR and LAMIN A/C as nuclear control for western blot. Gly acid glycyrrhetic acid.

Cortisol can be locally biosynthesized during decidualization in vitro^35^. Because MR has a similar affinity for aldosterone and cortisol, HSD11β2 can protect aldosterone binding to MR by inactivation of the high level of cortisol during decidualization^35^. Therefore, HSD11β2 inhibitor was used to block the nuclear translocation of aldosterone-stimulated MR^36^. Thus, we used HSD11β2 inhibitor glycyrrhetinic acid (30 μM) to restrain MR nuclear translocation. To well examine the active MR induced by aldosterone, 80 μM of aldosterone was used for further study. Immunofluorescence analysis showed that aldosterone-induced nuclear MR signal was reduced by glycyrrhetinic acid (Fig. 4a). Western blot analysis further verified that nuclear MR was decreased and cytoplasmic MR was increased by glycyrrhetinic acid (Fig. 4b). These results suggested that aldosterone stimulated nuclear MR level under the protection of HSD11β2.

Whether aldosterone benefits decidualization is still unknown. Thus, IGFBP1 and PRL mRNA levels were examined after aldosterone treatment. Both IGFBP1 and PRL mRNA levels were significantly increased by aldosterone, which was abrogated by glycyrrhetinic acid (Fig. 4c, d). FOXO1 is also a marker for human in vitro decidualization and has binding sites in the promoters of IGFBP-1 and PRL^1,37^. Both FOXO1 mRNA and nuclear FOXO1 levels were up-regulated by aldosterone, which was also suppressed by glycyrrhetinic acid (Fig. 4e, f).

FOXO1 is an important progesterone- and cAMP-dependent transcription factor in decidualizing hESCs^29^. Although FOXO1 is regulated by cAMP response element-binding protein (CREB)^38^, it is unknown how CREB regulates FOXO1 during human decidualization. Under in vitro decidualization, the protein levels of p-CREB and FOXO1 were significantly increased by aldosterone that was abrogated by glycyrrhetinic acid, while CREB did not change (Fig. 4f). KG-501, an inhibitor of CREB, was used to verify the regulation of FOXO1 by CREB. The nuclear levels of both p-CREB and FOXO1 were significantly reduced by KG-501 (40 μM) during decidualization (Fig. 4g). Moreover, the mRNA levels of IGFBP1, PRL and FOXO1 were also suppressed by KG-501 (Fig. S1a–c), indicating CREB should be at the upstream of FOXO1 during decidualization. All the results proposed that aldosterone could induce decidualization through p-CREB/FOXO1 signaling.

### PKD4 induction under decidualization

**Fig. 5:**
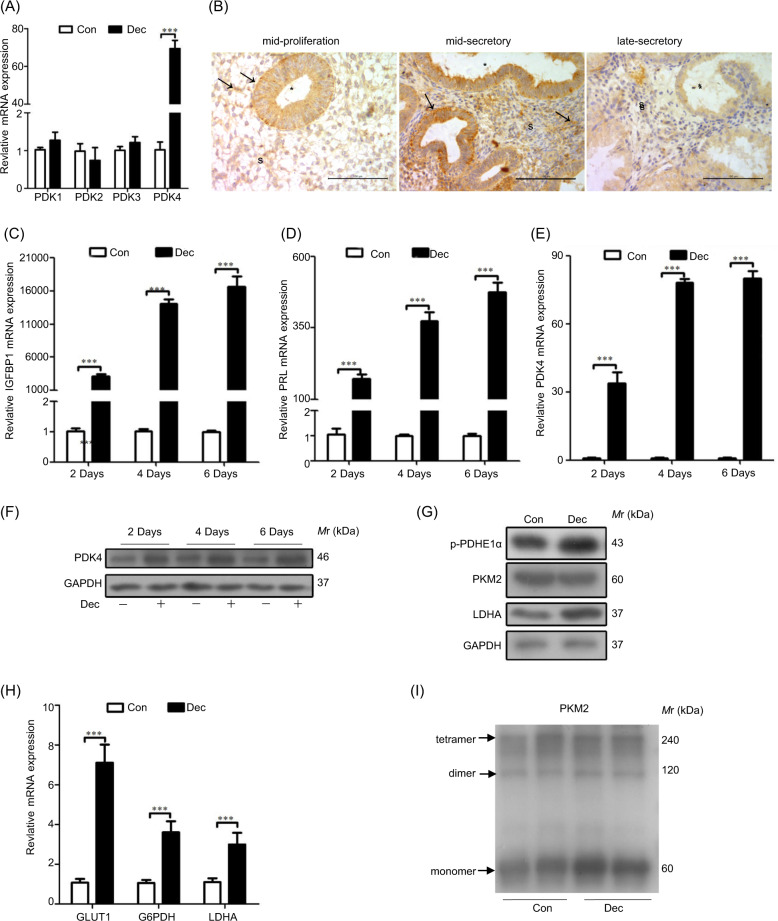
PDK4 expression and glycolysis during decidualization. **a** Real time PCR analysis of PDK1, PDK2, PDK3, and PDK4 during decidualization. **b** Immunohistochemical analysis of PDK4 protein in human endometrium during the menstrual cycle. Scale bar = 100 μm; s stromal cells; * glandular lumen. **c** IGFBP1 expression during decidualization; **d** PRL expression during decidualization. **e** PDK4 expression during decidualization. **f** Western blot analysis of PDK4 protein during decidualization. **g** Western blot analysis of p-PDHE1α, PKM2 and LDHA levels during decidualization. **h** RT-PCR analysis of GLUT1, G6PDH and LDHA mRNA expression during decidualization. **i** Western blot analysis of monomeric, dimeric and tetramer PKM2 proteins in decidual cells. RPL7 was used as internal control for real time PCR, and GAPDH as an internal control for Western blot.

In mice, there are an induction of glycolysis-related genes and a high level of lactate production in decidua, suggesting that Warburg-like glycolysis is activated^25^. But whether Warburg-like glycolysis is active during human decidualization is still unknown. Glucose transporter 1 (GLUT1), which is a pump for glucose uptake, is increased in human decidualizing cells^39^. PDK4 compromises PDC activity by phosphorylating p-PDHE1α, resulting in attenuation of pyruvate oxidation and promoting dehydrogenation of pyruvate^23^. There are 4 isoforms of PDK that can phosphorylate PDHE1α. Compared to controls, the expression levels of PDK1, PDK2, and PDK3 remained unchanged under in vitro decidualization, but PDK4 was significantly increased (Fig. 5a), suggesting that PDK4 should be the dominant form during decidualization. PDK4 immunostaining was mainly detected in glandular epithelium and weakly in stromal cells during mid-proliferative phase (Fig. 5b). PDK4 mRNA expression was significantly increased in stromal cells under in vitro decidualization for 2, 4 and 6 days, showing a similar pattern as IGFBP1 and PRL (Fig. 5c–e). Western blot showed PDK4 protein level was also up-regulated under in vitro decidualization (Fig. 5f).

p-PDHE1α, a subunit of PDC, can oxidative decarboxylate pyruvate and act as a surrogate marker of PDK activity^20^. p-PDHE1α, was also increased during decidualization (Fig. 5g). Under in vitro decidualization, glycolysis-related genes (GLUT1, G6PDH and LDHA) were also significantly elevated (Fig. 5g, h). PKM2 plays a critical role during Warburg glycolysis. The dimers and tetramers of PKM2 indicate the low and high levels of pyruvate kinase activity, respectively^40^. However, the levels for both dimers and tetramers of PKM2 remained unchanged during decidualization (Fig. 5g, i). These data suggested that Warburg effect should not be the dominant form of glycolysis during human decidualization.

PDK4 can inactivate PDC through phosphorylating PDHE1α, leading to acceleration of Warburg effect^20^. Therefore, silence of PDHE1α was used to compromise PDC activity. PDHE1α siRNA could effectively decrease PDHE1α mRNA expression (Fig. S2a, b). The inactivation of PDC led to down-regulation of IGFBP1 and PRL (Fig. S2c, d). But knockdown of PDHE1α had no effect on FOXO1 mRNA expression (Fig. S2b, e). These data suggested inactivation of PDC had adverse effects on decidualization through a non-FOXO1 pathway. Moreover, both mRNA and protein levels of LDHA did not change in siPDHE1α group (Fig. S2f), indicating inactivation of PDC has no effect on Warburg effect during decidualization.

### Silence of PDK4 results in down-regulation of p-CREB/FOXO1

**Fig. 6:**
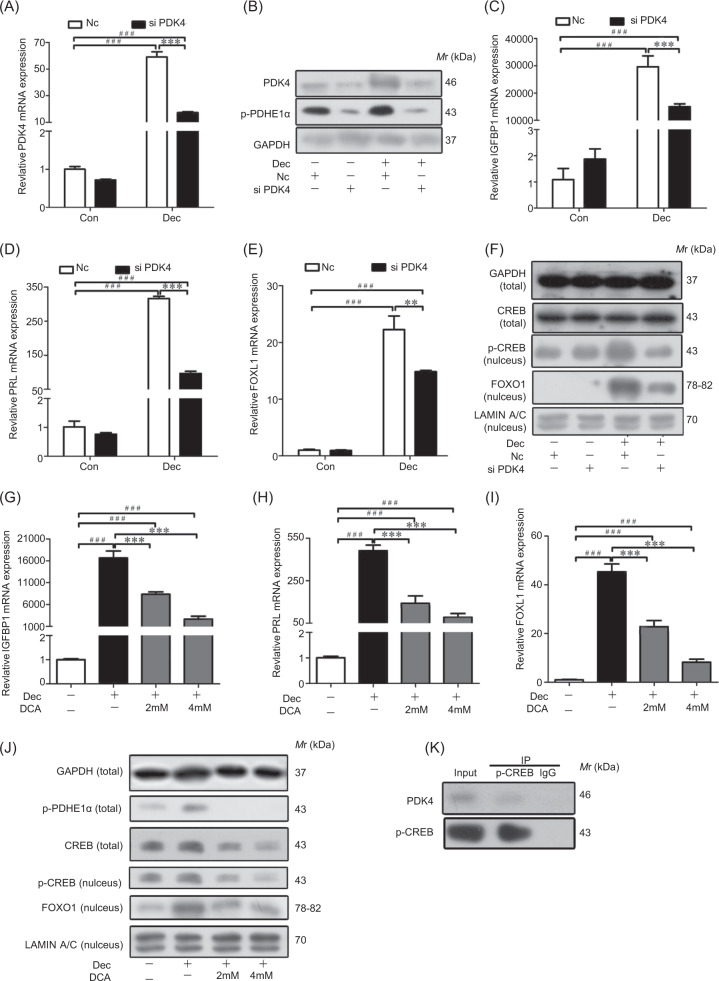
PDK4 function on decidualization. **a** PDK4 mRNA level in decidual cells transfected with control siRNA (NC) or PDK4 siRNA. **b** Western blot analysis of PDK4 and p-PDHE1α protein in decidual cells transfected with PDK4 siRNA. **c** IGFBP1 mRNA level after transfection with PDK4 siRNA. **d** PRL mRNA level after transfection with PDK4 siRNA. **e** FOXO1 mRNA level after transfection with PDK4 siRNA. **f** Western blot analysis of FOXO1 and p-CREB proteins in the nuclear fractions and CREB protein in decidual cells after transfection with PDK4 siRNA. **g** Effects of DCA on IGFBP1 mRNA level. **h** Effects of DCA on PRL mRNA level. **i** Effects of DCA on FOXO1 mRNA level. **j** Western blot analysis of p-PDHE1α, FOXO1 (nucleus), CREB, and p-CREB (nucleus) protein expression after decidual cells were treated with DCA. **k** The co-immunoprecipitation of PDK4 by p-CREB antibody in decidual cells. RPL7 was used as internal control for real time PCR, GAPDH as internal control and LAMIN A/C as nuclear control for western blot. Dichloroacetate (DCA), an inhibitor of all four PDK isoforms.

To further investigate the function of PDK4 on decidualization, siRNA was used to knockdown PDK4 in hESCs. After the levels of both PDK4 mRNA and protein were significantly reduced by PDK4 siRNA under decidualization (Fig. 6a, b), IGFBP1 and PRL mRNA levels were also remarkably suppressed by PDK4 knockdown (Fig. 6c, d). Furthermore, the mRNA and nuclear protein levels of FOXO1 were down-regulated by PDK4 siRNA during decidualization (Fig. 6e, f). Besides, p-CREB protein level was reduced by PDK4 siRNA (Fig. 6f).

Dichloroacetate (DCA), an inhibitor of all four PDK isoforms, should mainly inhibit PDK4 because PDK4 was the dominant form of PDK under decidualization in this study. Under in vitro decidualization, IGFBP1 and PRL mRNA levels were significantly inhibited by DCA (Fig. 6g, h). DCA treatment also reduced both the mRNA and nuclear protein levels of FOXO1 (Fig. 6i, j). Moreover, DCA treatment reduced both CREB and nuclear p-CREB in hESCs under in vitro decidualization (Fig. 6j).

Because PDK4 can bind with CREB and prevent its degradation in brain^41^, co-immunoprecipitation was used to examine whether PDK4 can interact with p-CREB. PDK4 was immunoprecipitated by p-CREB antibody (Fig. 6k), indicating that PDK4 might have a physical interaction with CREB.

### AMPK acts at the upstream of PDK4 in decidual cells

**Fig. 7:**
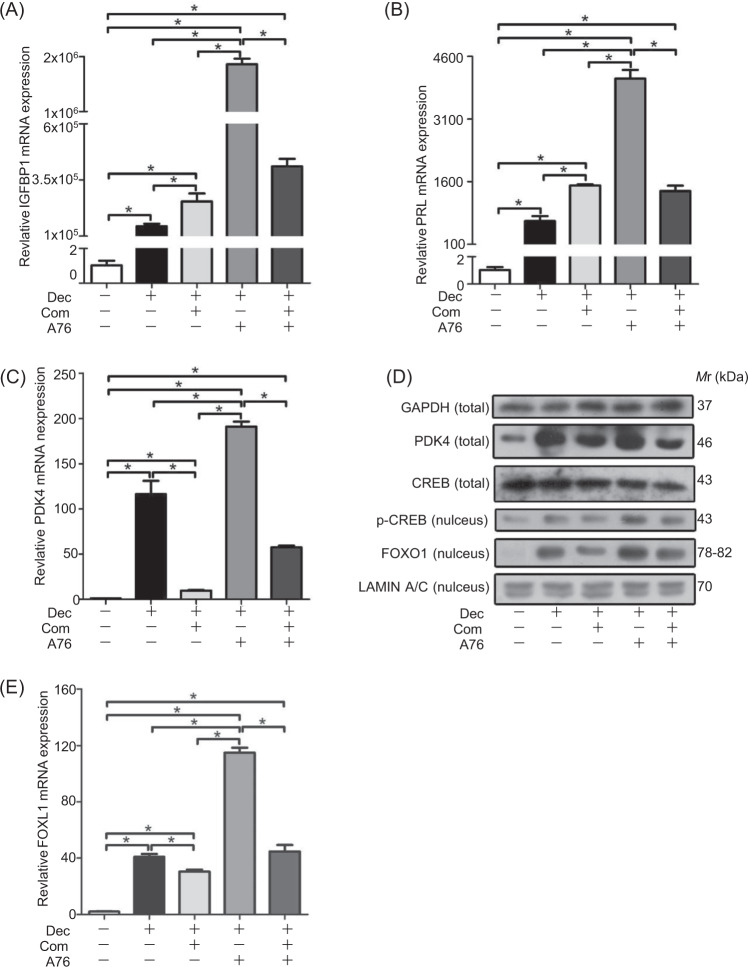
Effects of AMPK on decidualization. **a** Effects of A76 and Compound C on IGFBP1 expression. **b** Effects of A76 and Compound C on PRL expression. **c** Effects of A76 and Compound C on PDK4 expression. **d** Western blot analysis of PDK4, FOXO1(nucleus), CREB, and p-CREB (nucleus) protein expression after decidual cells were treated with A76 or Compound C. **e** Effects of A76 and Compound C on FOXO1 expression. RPL7 was used as internal control for real time PCR, GAPDH as internal control and LAMIN A/C as nuclear control for western blot. A-769662 (A 76), AMPK activator; Compound C (Com), AMPK inhibitor.

AMP-activated protein kinase (AMPK) acts as a regulator of PDK4 in muscle and cardiomyocytes^42,43^. However, it is unknown whether AMPK is essential for induction of PDK4 during decidualization. After stromal cells were treated with A76 (20 μM), a p-AMPK activator that displays selectivity towards β1 subunit-containing heterotrimers, IGFBP1 and PRL mRNA expression was markedly increased. Compound C (5 μM), a p-AMPK inhibitor, also slightly stimulated the expression of IGFBP1 and PRL (Fig. 7a, b). However, the induction of IGFBP1 and PRL by A76 was abrogated by Compound C (Fig. 7a, b). PDK4 mRNA and protein levels were stimulated by A76, but significantly down-regulated by Compound C (Fig. 7c, d). A76-stimulated PDK4 increase was suppressed by Compound C, suggesting that PDK4 was regulated by p-AMPK. FOXO1 expression showed a similar pattern as PDK4 after decidual cells were treated with A76 or Compound C (Fig. 7d, e). Likewise, p-CREB was also stimulated by A76, and inhibited by Compound C (Fig. 7e).

### Aldosterone induces LKB1 expression to activate p-AMPK through binding MR

Liver kinase B1 (LKB1) can phosphorylate Thr172 at the α subunit of AMPK^44^. Based on our analysis on the LKB1 promoter by JASPAR, there was a MR binding site in the LKB1 promoter. LKB1 was stimulated by aldosterone, which was abrogated by glycyrrhetinic acid (Fig. S3a). Furthermore, aldosterone treatment induced the increase of p-AMPK and PDK4 in decidual cells, which was abrogated by glycyrrhetinic acid (Fig. S3a, b). These data suggested that aldosterone might activate LKB1/p-AMPK/PDK4 signaling through MR during decidualization.

### Aldosterone regulates decidualization through p-AMPK/PDK4/p-CREB/FOXO1

**Fig. 8:**
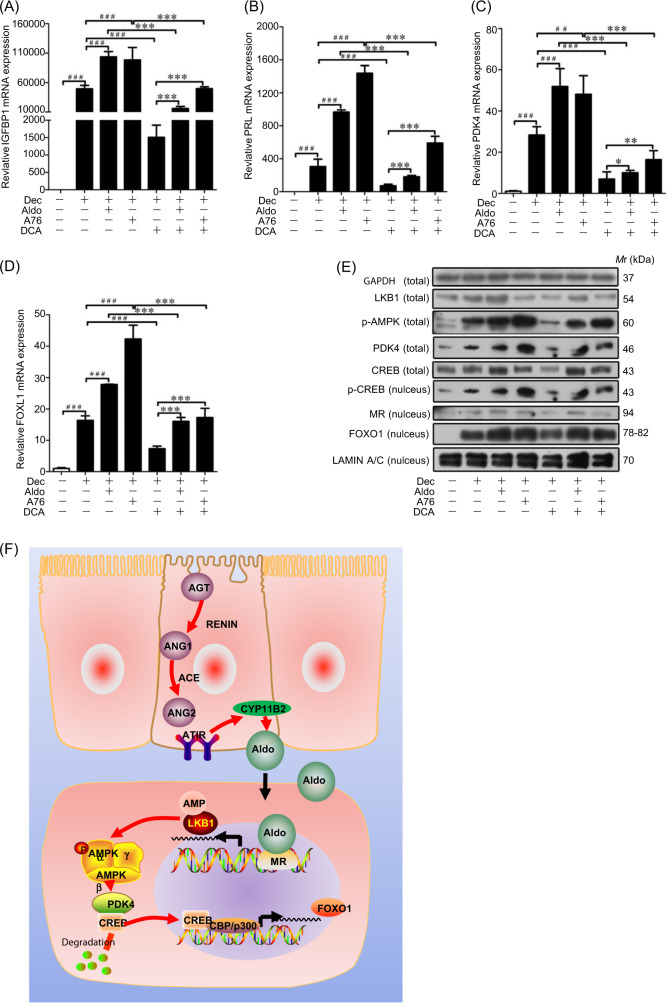
Effects of aldosterone, A76 and DCA on decidualization. **a** Effects of aldosterone, A76 and DCA on IGFBP1 expression. **b** Effects of aldosterone, A76 and DCA on PRL expression. **c** Effects of aldosterone, A76 and DCA on PDK4 expression. **d** Effects of aldosterone, A76 and DCA on FOXO1 expression. **e** Western blot analysis of LKB1, p-AMPK, PDK4, FOXO1, CREB, and p-CREB (nucleus) protein levels after decidual cells were treated with aldosterone, A 76 and DCA, respectively. GAPDH as internal control and LAMIN A/C as nuclear control. **f** A schematic pathway showing the regulation of glandular aldosterone on stromal decidualization through p-AMPK/PDK4/p-CREB/FOXO1 pathway. RPL7 was used as internal control for real time PCR, GAPDH as internal control and LAMIN A/C as nuclear control for Western blot. A-769662 (A 76), AMPK activator; Compound C (Com), AMPK inhibitor; dichloroacetate (DCA), an inhibitor of all four PDK isoforms.

In decidual cells, IGFBP1 and PRL mRNA levels were induced by aldosterone or A76, but extremely suppressed by DCA (Fig. 8a, b). The mRNA levels of FOXO1 and PDK4, and the nuclear protein levels of FOXO1 and p-CREB were significantly increased by aldosterone or A76 (Fig. 8c–e). A schematic pathway was proposed to show how glandular epithelium-derived aldosterone regulates human decidualization via LKB1-AMPK-PDK4-CREB-FOXO1 pathway (Fig. 8f).

## Discussion

RAS is important for regulating electrolytes and blood pressure^45^. Recently, the expression of local RAS has been reported in human female reproductive system^46^. Activation of ACE2/ANG 1-7/Mas axis is known as a regulator of placentation and angiogenesis of fetal development in rodents^46^. ANG2-ATR-pathway acts as a modulator of vascular function and angiogenesis in the ovary^46^. However, the studies about functional relevance of RAS in the uterus are limited. AGT protein is expressed in the glandular epithelium, stroma, and perivascular region in human endometrium^47^. Furthermore, ANG 2 level in plasma also elevates during early gestation^48^. The expression of RENIN and ACE are located in endometrial glandular epithelium throughout the menstrual cycle^18^. Our immunohistochemical data verified the expression of RENIN and ACE in glandular epithelium during mid-proliferative and mid-secretory phases. It is reported that ANG 2 level in uterine glands during mid-proliferation phase is similar to mid-secretory phase^19^. Moreover, a high level of AT1R mRNA expression is detected in glandular epithelium in secretory endometrium^19^. We found that AT1R protein level in uterine glands is elevated in mid-secretory phase compared with mid-proliferative phase, indicating ANG 2 may act by binding to AT1R. AT1R is associated with induction of CYP11B2 and responsible for synthesis of aldosterone^12^. In mice, deficiency of aldosterone caused by CYP11B2 knockout impairs placental function, leading to fetus loss or smaller pups^49^. However, whether CYP11B2 is expressed in human endometrium is not known. In our study, CYP11B2 is expressed in glandular epithelium during mid-secretory phase, and ANG2 can stimulate CYP11B2 expression through binding to AT1R in Ishikawa and ECC1 cells. However, aldosterone is not secreted in these cells. Progesterone, as a precursor of aldosterone, is increased during secretory phase and required for pregnancy survival andstimulates aldosterone release in adrenocortical cells in vitro^1,50^. In our results, progesterone can activate RAAS and stimulate aldosterone release in endometrial epithelial cells. It is reported that urinary and serum aldosterone levels are significantly higher during secretory phase^51^. Thus, RAAS should be activated in endometrial glands during secretory phase, leading to the synthesis of aldosterone.

The high level of serum aldosterone in healthy pregnancy benefits for expansion of physiological plasma volume^52^. A low level of serum aldosterone is common to preeclampsia, suggesting the dysfunction of RAAS may be responsible for pathophysiology of preeclampsia^53^. Defective or deficient decidualization increases the risk for developing preeclampsia^7,54^. The strong expression of CYP11B2 in glandular epithelium in pregnant decidua suggests that aldosterone should be mainly released from glands of pregnant decidua. However, the function of aldosterone synthesized in endometrial glands remains unknown.

The strong expression of MR in endometrial stroma during mid-secretory phase suggests that aldosterone may have a function during decidualization. MR expression is increased in secretory endometrium and first-trimester decidua^55^. Decidualization triggers a reciprocal increase in MR protein level, and a decrease in glucocorticoid receptor (GR) expression^35^. MR and GR show a significant amino acid homology in their ligand- and DNA-binding domains. However, their expression pattern is different during human decidualization^35,56^. Although GR expression is increased during early pregnancy and is critical for mouse fertility, GR decreases in human decidual cells. GR signaling plays a role in chromatin remodeling during human decidualization^35^. Human MR binds cortisol and aldosterone with a similar affinity, whereas HSD11β2 is able to successfully exclude glucocorticoids from MR via the rapid inactivation of cortisol into inert cortisone^57^. In addition, HSD11β2 functionally interacts with MR and directly regulates the nuclear translocation of MR induced by aldosterone^58^. The nuclear translocation of aldosterone-stimulated MR is blocked by a strict HSD11β2-dependent mechanism^36^. Human HSD11β2 is located in the functional layer of first trimester decidua, and its protein level is also increased during *in vitro* decidualization^55,35^. Because progesterone drives the local biosynthesis of cortisol in decidual cells, HSD11β2 inhibitor is used to prevent aldosterone-induced MR activation in this study. We found that aldosterone-induced decidualization is inhibited after the nuclear translocation of MR is prevented, indicating that aldosterone function on decidualization depends on MR activation and is regulated by HSD11β2. These data indicate that aldosterone may have a function on decidualization through binding to MR.

MR mediates the actions of two important hormones, aldosterone and cortisol. However, its roles in early pregnancy are still not clear. MR-deficient mice die in 1–2 weeks postnatally due to salt wasting and hyperkalaemia, following elevated plasma renin and aldosterone^59^. Under decidualization in vitro, aldosterone can induce the rapid nuclear translocation of MR. We found that aldosterone treatment can stimulate IGFBP1 and PRL mRNA levels through FOXO1, which can be activated by p-CREB. However, it is unknown whether FOXO1 can be regulated by p-CREB in decidual cells. The suppression of FOXO1 expression by CREB inhibitor demonstrates that p-CREB acts as a regulator of FOXO1 during decidualization. Our data suggested aldosterone promotes decidualization through p-CREB/FOXO1 pathway by activating MR.

Pyruvate dehydrogenase complex (PDC) is inactivated by PDKs via phosphorylating PDHE1α^20^. PDK4, one of four PDKs, is induced by PGC-1α during human decidualization in vitro^60^. Our study showed that PDK4 is increased in human endometrial glands and stroma during mid-secretory phase, and strongly induced during decidualization in vitro. Inhibition of PDK4 suppresses decidualization through FOXO1. However, knockdown of PDHE1α has no effect on decidualization and on FOXO1 expression. Moreover, Warburg-effect is not detected during decidualization in our study. The high levels of GLUT1 and G6PDH during decidualization may be responsible for pentose phosphate pathway, which is essential for human decidualization in vitro^45^. These data indicate that putative PDK4 suppression of PDHE1α does not contribute to decidualization. It is reported that PDK4 can physically interact with p-CREB to prevent its degradation in tumor^60^. The co-immunoprecipitation between PDK4 and CREB in decidual cells suggests that PDK4 may interact with CREB to prevent the degradation of CREB.

During in vitro decidualization, activation of AMPK can induce PDK4 expression, followed by the stimulation of p-CREB/FOXO1 pathway. Moreover, PDK4 and p-CREB/FOXO1 pathway is suppressed by AMPK knockdown. These results imply that the activation of AMPK has a positive regulation on PDK4 during human decidualization. LKB1 is one of the important mediators for activation of AMPK^61^. According to promoter prediction, MR has a binding site in the promoter of LKB1. In our study, aldosterone may induce LKB1 expression to activate AMPK through binding MR.

RAAS is vital for fluid/electrolyte homeostasis and arterial blood pressure^9^. The exact function of aldosterone on human endometrium is poorly defined. During the mid-secretory phase of menstrual cycle, aldosterone is synthesized in glandular epithelium through activation of RAAS. Paracrine aldosterone induces LKB1 by binding to its MR. LKB1-induced AMPK phosphorylation can activate PDK4 to prevent the degradation of p-CREB. Altogether, we have not only taken steps to increase the current understanding on aldosterone synthesis in endometrial glands, but also determined the role of aldosterone in decidualization. Since decidualization is a complex process, AMPK/PDK4/p-CREB/FOXO1 pathway may not be the only target of aldosterone during human decidualization. Aldosterone mainly involves in regulation of blood pressure, and can activate specific intracellular genomic and nongenomic pathways^62^. Here, we only reveal the genomic pathway regulated by aldosterone during decidualization. Furthermore, epithelial sodium channel (ENaC) and serum and glucocorticoid inducible kinase (SGK1) are considered the final effectors of Aldo/MR system in the kidney^62^. Aberrant regulation of SGK1 and ENaC in uterine epithelium could contribute to the pathogenesis of unexplained infertility in humans and mice^63,64^. We believe that aldosterone not only involves in decidualization, but also other processes during pregnancy. In this study, our data provide a new insight into decidualization by uncovering an instructive role for aldosterone in regulating decidualization.

## Supplementary information

Supplemental Figure and Table legends

Figure S1

Figure S2

Figure S3

Supplemental Table 1
