# Supplementary material for: Aldosterone from endometrial glands is benefit for human decidualization
Source: Cell Death Dis. 2020 Aug 13;11(8):679. doi: 10.1038/s41419-020-02844-9 (PMC7442827; doi:10.1038/s41419-020-02844-9)
Supplement: Supplementary file 1 — Supplemental Figure and Table legends [file 41419_2020_2844_MOESM1_ESM.docx]

Supplemental figure and table legends

Figure S1 Effects of KG-501, an inhibitor of CREB, on human in vitro decidualization. (A) IGFBP1 mRNA levels after KG-501 treatment. (B) PRL mRNA levels after KG-501 treatment. (C) FOXO1 mRNA levels after KG-501 treatment.

Figure S2 Effects of PDHE1α on human in vitro decidualization. (A) PDHE1α mRNA levels in stromal cells and decidual cells transfected with control siRNA (NC) or PDHE1α siRNA. (B) Western blot analysis of PDHE1α, FOXO1 and LDHA proteins in decidual cells after transfection with PDHE1α siRNA. (C) IGFBP1 mRNA levels after transfection with PDHE1α siRNA. (D) PRL mRNA levels after transfection with PDK4 siRNA. (E) FOXO1 mRNA levels after transfection with PDHE1α siRNA. (F) LDHA mRNA levels after transfection with PDHE1α siRNA.

Figure S3 Effects of aldosterone and glycyrrhetinic acid (an inhibitor for HSD11β2) on LKB1, p-AMPK and PDK4 under human in vitro decidualization. (A) Western blot analysis of LKB1, p-AMPK and PDK4 proteins after cells were treated with aldosterone and glycyrrhetinic acid. (B) Real time PCR analysis of PDK4 mRNA level after cells were treated with aldosterone and glycyrrhetinic acid. Aldo, aldosterone; Dec, in vitro decidualization; Gly acid, glycyrrhetinic acid.

Table S1 Primers used in this study.
