# Supplementary figures and images for: Aldosterone from endometrial glands is benefit for human decidualization
Source: Cell Death Dis. 2020 Aug 13;11(8):679. doi: 10.1038/s41419-020-02844-9 (PMC7442827; doi:10.1038/s41419-020-02844-9)

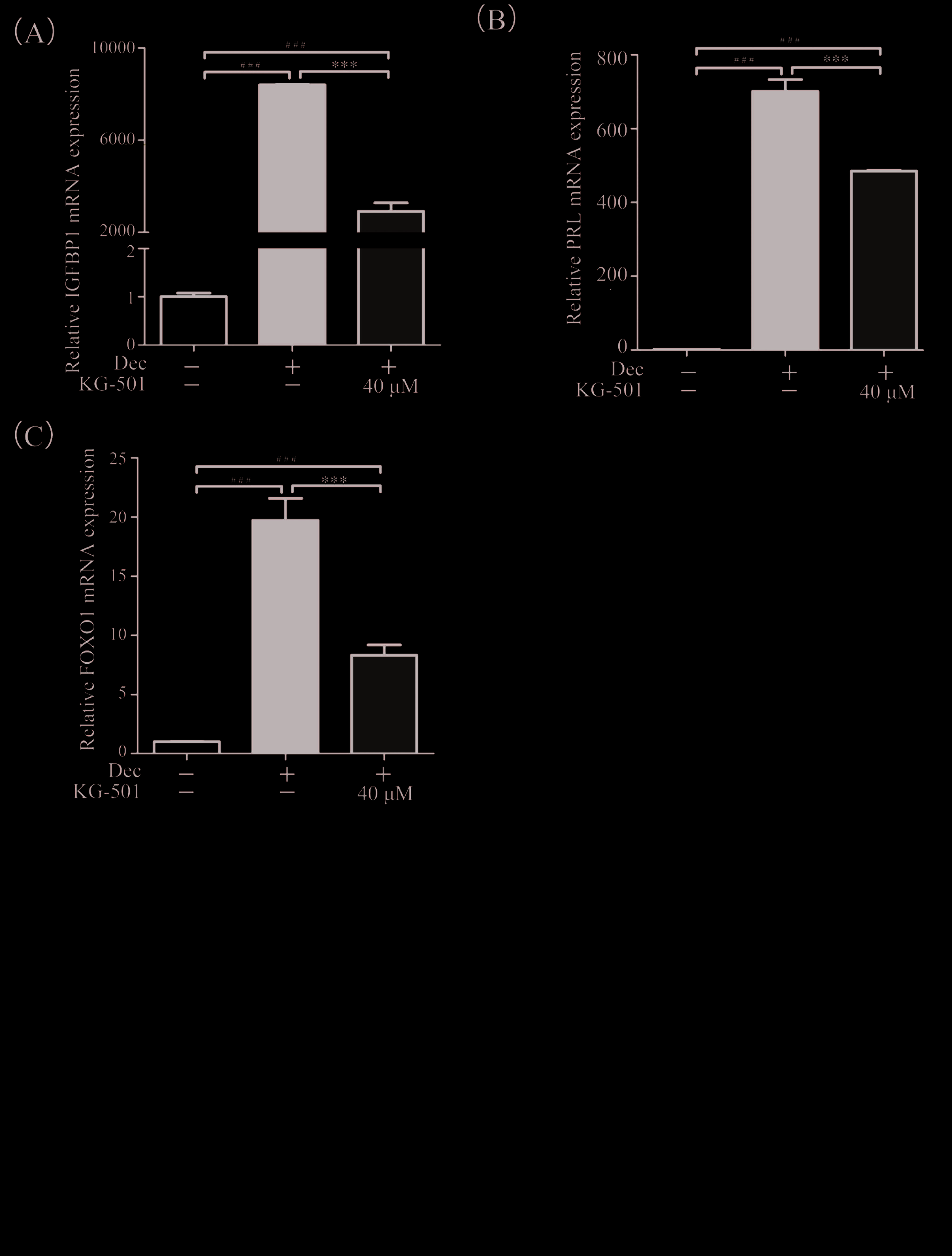

Supplement: Supplementary file 2 — Figure S1 [file 41419_2020_2844_MOESM2_ESM.tif]

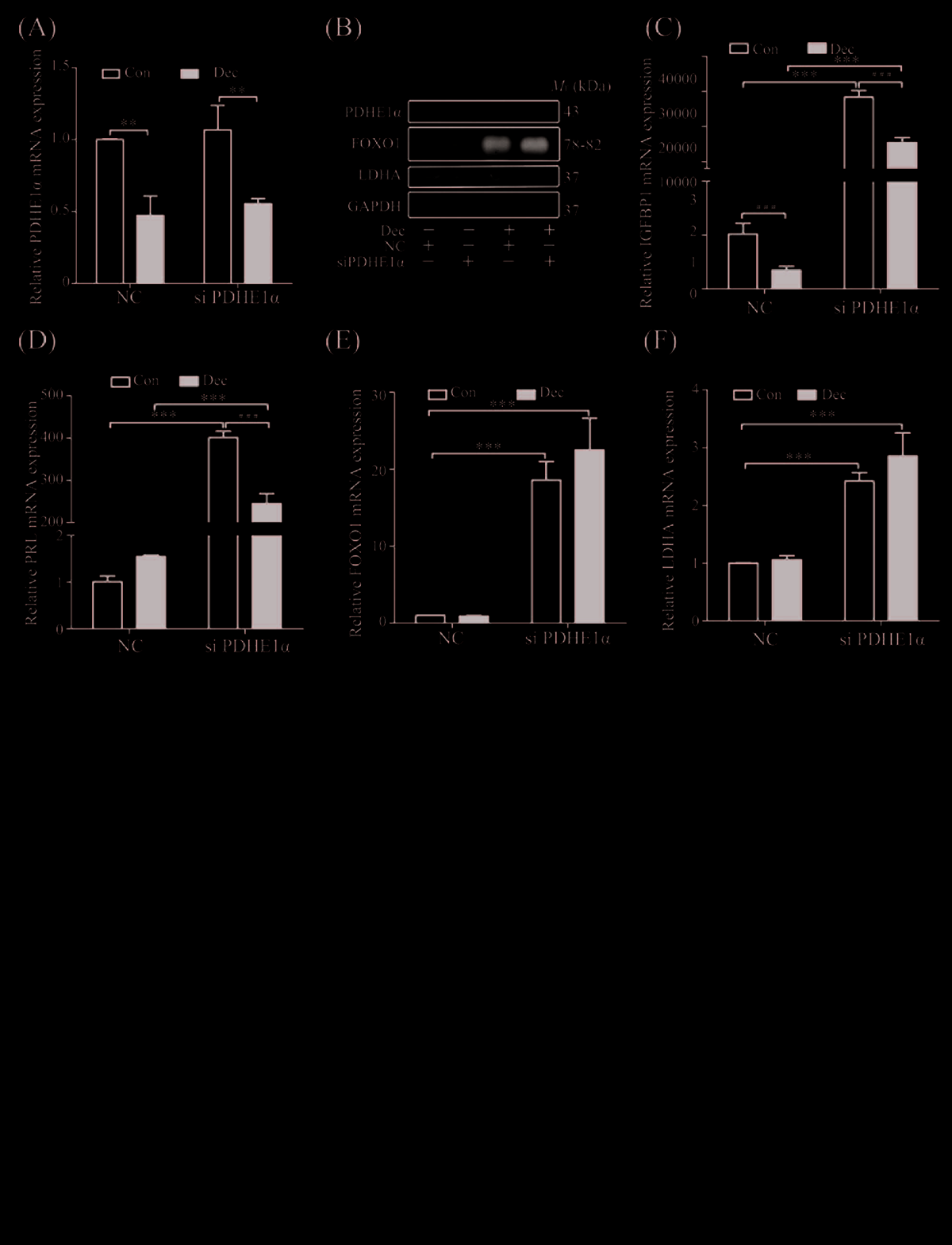

Supplement: Supplementary file 3 — Figure S2 [file 41419_2020_2844_MOESM3_ESM.tif]

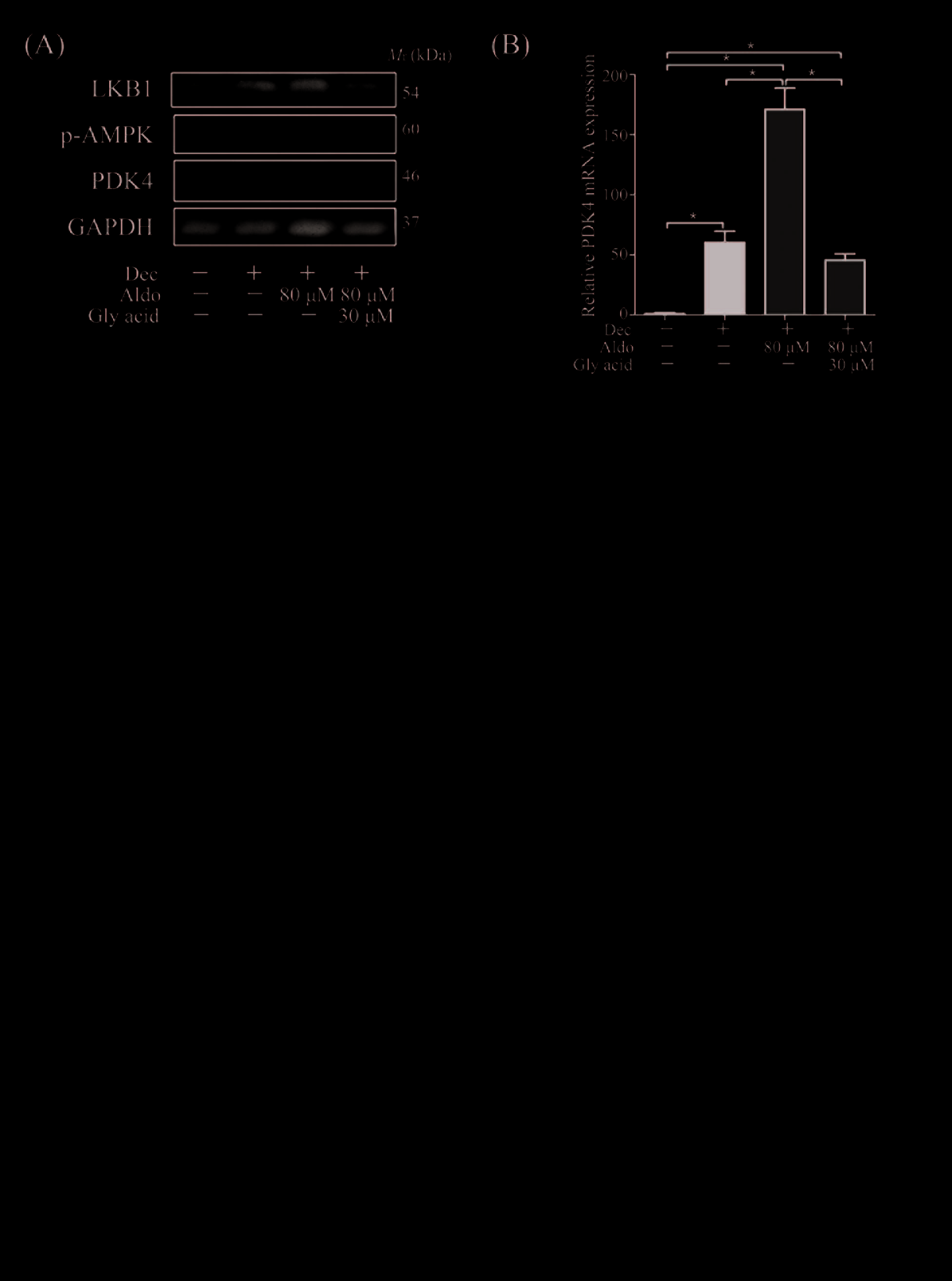

Supplement: Supplementary file 4 — Figure S3 [file 41419_2020_2844_MOESM4_ESM.tif]
