# Supplementary material for: Aldosterone from endometrial glands is benefit for human decidualization
Source: Cell Death Dis. 2020 Aug 13;11(8):679. doi: 10.1038/s41419-020-02844-9 (PMC7442827; doi:10.1038/s41419-020-02844-9)
Supplement: Supplementary file 5 — Supplemental Table 1 [file 41419_2020_2844_MOESM5_ESM.docx]

Table S1. Primers used in this study

| Primers | Sequences 5’ to 3’ | Accession number |
| --- | --- | --- |
| AMPKa1 | ACCACAACCGCTCTAGTCTAA  CCACATCAAGGCTCCGAATC | [NM_001355028.1](https://www.ncbi.nlm.nih.gov/nuccore/NM_001355028.1) |
| AMPKa2 | GTGGTGTTATCTTGTATGCTCTTC  TGGCGACAGAACGATTGAGA | [NM_006252.4](https://www.ncbi.nlm.nih.gov/nuccore/NM_006252.4) |
| FOXO1 | CGAGCTGCCAAGAAGAAA  TTCGAGGGCGAAATGTAC | NM_002015 |
| G6PDH | CTGTGCGAGCCGTGCG  CGGGTCTGAGAGTGGG | NM_001282587.1 |
| GLUT1 | AGAGGTTATGTGCCTGAAGTCG  GGGTGAAGGAGGAGGATGAG | NM_006516.2 |
| IGFBP1 | CCAAACTGCAACAAGAATG  GTAGACGCACCAGCAGAG | NM_001013029 |
| LDHA | AACCGTGTTATTGGAAGCG  GAGACACCAGCAACATTCATTC | NM_001135239.1 |
| PDHE1α | TGGAAGTGAGGAAGGAGAT  GTCGCTGGAGTAGATGTG | [NM_000284.3](https://www.ncbi.nlm.nih.gov/nuccore/NM_000284.3) |
| PDK1 | GGATGTGAATGGGCAGTTAG  AAGGAATAGTGGGTTAGGTGAG | NM_001278549.1 |
| PDK2 | GTGGAGTCAAGCCGCCTAT  CTGAGGAAGGTGAAGGAGGTT | [NM_001199898.1](https://www.ncbi.nlm.nih.gov/nuccore/NM_001199898.1) |
| PDK3 | GCCTACTGCCTGACTCTGAC  TGCTTGTGAGGATGTGGTGAT | NM_001142386.2 |
| PDK4 | TCCTAACTGTGATGTGGTAGCA  ACGATGTGAATTGGTTGGTCTG | [NM_002612.3](https://www.ncbi.nlm.nih.gov/nuccore/NM_002612.3) |
| PRL | AAGCTGTAGAGATTGAGGAGCAAA  TCAGGATGAACCTGGCTGACTA | NM_000948 |
| RPL7 | GCAGATGTACCGCACTGAGATTC  ACCTTTGGGCTTACTCCATTGATA | NM_000948 |
